# Independent *DSG4* frameshift variants in cats with hair shaft dystrophy

**DOI:** 10.1007/s00438-021-01842-6

**Published:** 2021-12-08

**Authors:** Sarah Kiener, Ana Rostaher, Silvia Rüfenacht, Vidhya Jagannathan, John P. Sundberg, Monika Welle, Tosso Leeb

**Affiliations:** 1 Institute of Genetics, Vetsuisse Faculty, University of Bern, 3001 Bern, Switzerland; 2 University of Bern, 3001 Bern, Switzerland; 3 Clinic for Small Animal Internal Medicine, Vetsuisse Faculty, University of Zurich, 8057 Zurich, Switzerland; 4 DermaVet, Tierklinik Aarau West, 5036 Oberentfelden, Switzerland; 5 The Jackson Laboratory, Bar Harbor, ME 04609 USA; 6 Institute of Animal Pathology, Vetsuisse Faculty, University of Bern, 3001 Bern, Switzerland

**Keywords:** *Felis catus*, Whole-genome sequencing, Dermatology, Genodermatosis, Alopecia, Lanceolate, Hair shaft dysplasia

## Abstract

Investigations of hereditary phenotypes in spontaneous mutants may help to better understand the physiological functions of the altered genes. We investigated two unrelated domestic shorthair cats with bulbous swellings of the hair shafts. The clinical, histopathological, and ultrastructural features were similar to those in mice with lanceolate hair phenotype caused by loss-of-function variants in *Dsg4* encoding desmoglein 4. We sequenced the genomes from both affected cats and compared the data of each affected cat to 61 control genomes. A search for private homozygous variants in the *DSG4* candidate gene revealed independent frameshift variants in each case, c.76del or p.Ile26fsLeu*4 in case no. 1 and c.1777del or p.His593Thrfs*23 in case no. 2. DSG4 is a transmembrane glycoprotein located primarily in the extracellular part of desmosomes, a complex of adhesion molecules responsible for connecting the keratin intermediate filaments of neighbouring epithelial cells. Desmosomes are essential for normal hair shaft formation. Both identified *DSG4* variants in the affected cats lead to premature stop codons and truncate major parts of the open-reading frame. We assume that this leads to a complete loss of DSG4 function, resulting in an incorrect formation of the desmosomes and causing the development of defective hair shafts. Together with the knowledge on the effects of *DSG4* variants in other species, our data suggest that the identified *DSG4* variants cause the hair shaft dystrophy. To the best of our knowledge, this study represents the first report of pathogenic *DSG4* variants in domestic animals.

**Supplementary Information:**

The online version contains supplementary material available at 10.1007/s00438-021-01842-6.

## Introduction

Hair is composed of terminally differentiated dead keratinocytes. It is characteristic for terrestrial mammals, and involved in various functions such as physical protection, thermal regulation, or sensory perception. The hair follicle, a complex miniorgan residing in the dermal layer of the skin, is responsible for the formation of hair (Schneider et al. 2009). The hair shaft consists of the hair cuticle, the cortex, and the innermost medulla. It is surrounded by the inner root sheath, the companion layer, and the outer root sheath (Cheng and Bayliss 2008; Shimomura and Christiano 2010; Welle and Wiener 2016).

Significant progress has been made in identifying numerous genes expressed in the hair follicle (Shimomura and Christiano 2010; Wiener et al. 2020). A malfunctioning hair follicle, e.g., due to a genetic defect, represents one of several possible causes for hair loss resulting in alopecia or hypotrichosis (Cheng and Bayliss 2008; Schneider et al. 2009; Shimomura and Christiano 2010; Welle and Wiener 2016; Ahmed et al. 2019).

Targeted or spontaneous mutant mice provided many valuable resources for dermatological research (Sundberg 1994; Sundberg and King 1996, 2000; Nakamura et al. 2001; Chen et al. 2008). An ethylnitrosourea induced and, subsequently, a spontaneous mouse mutant with irregular hair shafts characterized by focal deformities and pronounced enlargement at the apex, resembling a lance head, were termed lanceolate hair (*lah* and *lah-J*) (Montagutelli et al. 1996; Sundberg et al. 2000). The causative genetic variant for the *lah* mouse mutant was identified in the *Dsg4* gene encoding desmoglein 4 (Kljuic et al. 2003). Several additional spontaneous allelic mutants with the same phenotype have been discovered in mice (Berry and Sundberg, unpublished data). A related phenotype in humans, localized autosomal recessive hypotrichosis (LAH, OMIM #607903), is due to genetic variants in the human ortholog, *DSG4* (Kljuic et al. 2003). Finally, a spontaneous lanceolate hair phenotype due to a *Dsg4* variant also exists in rats (Jahoda et al. 2004).

We previously described the clinical and histopathological findings in a litter of cats with alopecia and hair shaft irregularities that closely resembled the phenotype of *Dsg4*-deficient *lah* mice (Rostaher et al. 2021). In the present study, we investigated one additional unrelated case of a similar hair shaft dystrophy and searched for the underlying causative genetic variants in the previously reported cats.

## Methods

### Animal selection

This study included two unrelated domestic shorthair (DSH) cats affected by hair shaft dystrophy, and 61 genetically diverse control cats. The 63 sequenced cats included 34 purebred cats from 11 different breeds and 29 random-bred domestic cats (Table S1). Genomic DNA was isolated from ETDA blood samples with the Maxwell RSC Whole Blood Kit using a Maxwell RSC instrument (Promega, Dübendorf, Switzerland).

### Histopathological examinations

Skin biopsies from alopecic skin were taken under general anesthesia according to standard procedures. The biopsies were processed routinely and stained with hematoxylin and eosin, prior to histological evaluation.

### Whole-genome sequencing

Illumina TruSeq PCR-free DNA libraries with ~ 300 bp insert size of both affected cats were prepared and sequenced at 24 × coverage (case no. 1) and 21 × coverage (case no. 2) on a NovaSeq 6000 instrument. The reads were mapped to the FelCat9.0 cat reference genome assembly as described (Jagannathan et al. 2019). The sequence data were submitted to the European Nucleotide Archive with the study accession PRJEB7401 and sample accessions SAMEA7853384 (case no. 1) and SAMEA7853385 (case no. 2).

### Variant calling and filtering

Variant calling was performed as described (Jagannathan et al. 2019). The SnpEff software was used together with NCBI annotation release 104 of the FelCat9.0 genome reference assembly to predict the functional effects of the called variants (Cingolani et al. 2012). For variant filtering, we used 61 control genomes (Table S1). Filtering was done with a self-written C +  + program on a modified vcf-file containing genotype calls of the 2 affected cats and the 61 control cats, and SnpEff predicted functional effects of all variants.

### Gene analysis

Numbering within the feline *DSG4* gene corresponds to the NCBI RefSeq accession numbers XM_019815116.1 (mRNA) and XP_019670675.1 (protein).

### Sanger sequencing

Sanger sequencing of PCR amplicons was used to confirm the candidate variants *DSG4*:c.76del and *DSG4*:c.1777del and to genotype cats. We used forward and reverse primers together with the AmpliTaqGold360Mastermix (Thermo Fisher Scientific, Waltham, MA, USA) to amplify a PCR product from genomic DNA (Table 1). After treatment with shrimp alkaline phosphatase and exonuclease I, PCR amplicons were sequenced on an ABI 3730 DNA Analyzer (Thermo Fisher Scientific). The Sequencher 5.1 software was used to analyze the Sanger sequences (GeneCodes, Ann Arbor, MI, USA).

**Table 1:** Details of the primers used for Sanger sequencing

Variant	Primer name	Primer sequence (5′ to 3′)	Amplicon length (bp)	*T*_*M*_ (°C)
*DSG4*:c.76del	Primer F1	GGAGGGCAAAGAGCCTGTAT	362	60
	Primer R1	TGGGTTTGCCATTGCTATTT		
*DSG4*:c.1777del	Primer F2	GACGGCTGAGCAGCTTTTAT	445	60
	Primer R2	GCCCTTATTAGCCCCATTGT		

## Results

### Family anamnesis, clinical examinations, and histopathology

**Fig. 1:**
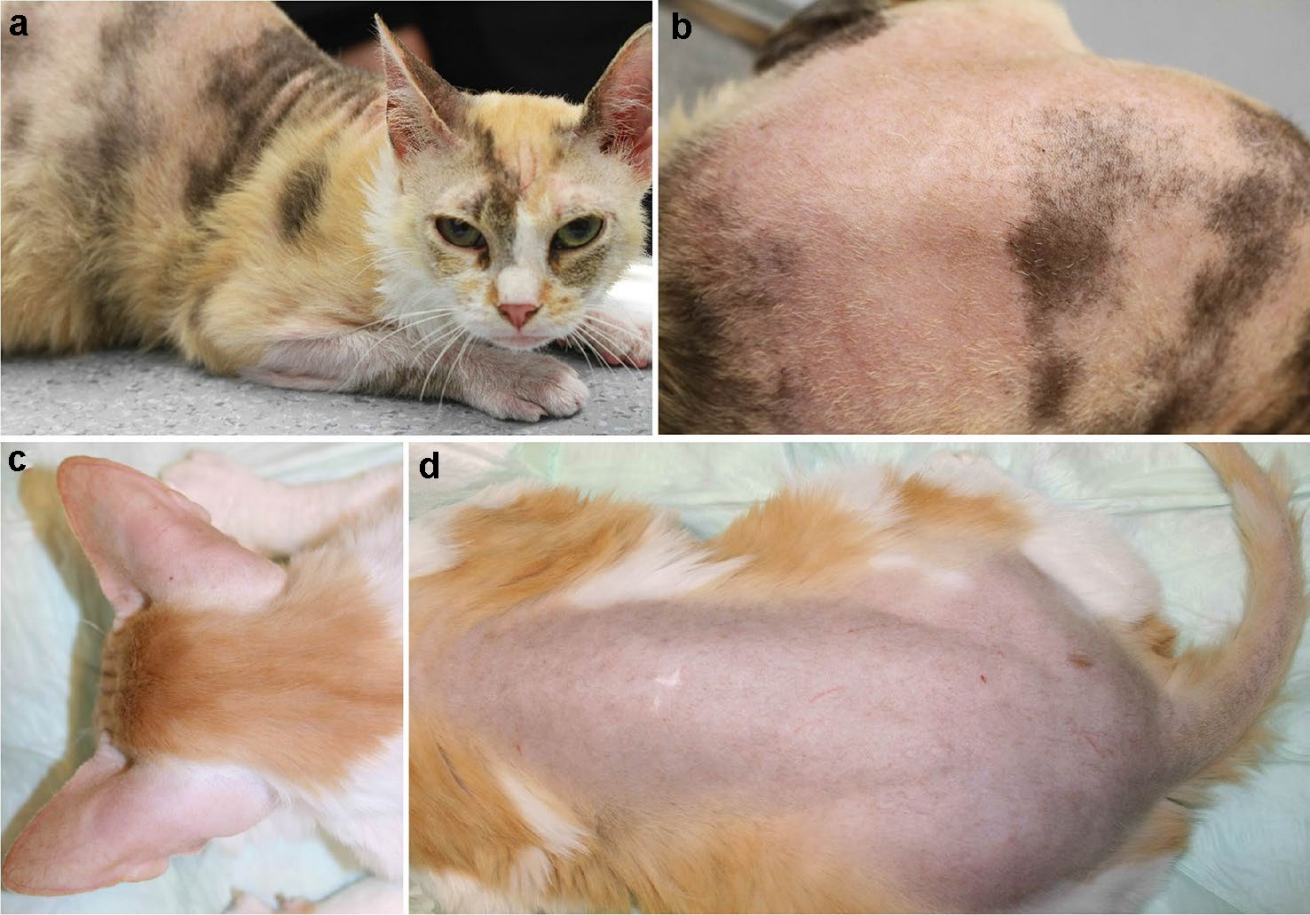
Clinical phenotype of the affected cats with hypotrichosis and alopecia on the convex pinnae, parts of the face, the back, and the legs. **a**, **b** Case no. 1. **c**, **d** Case no. 2

**Fig. 2:**
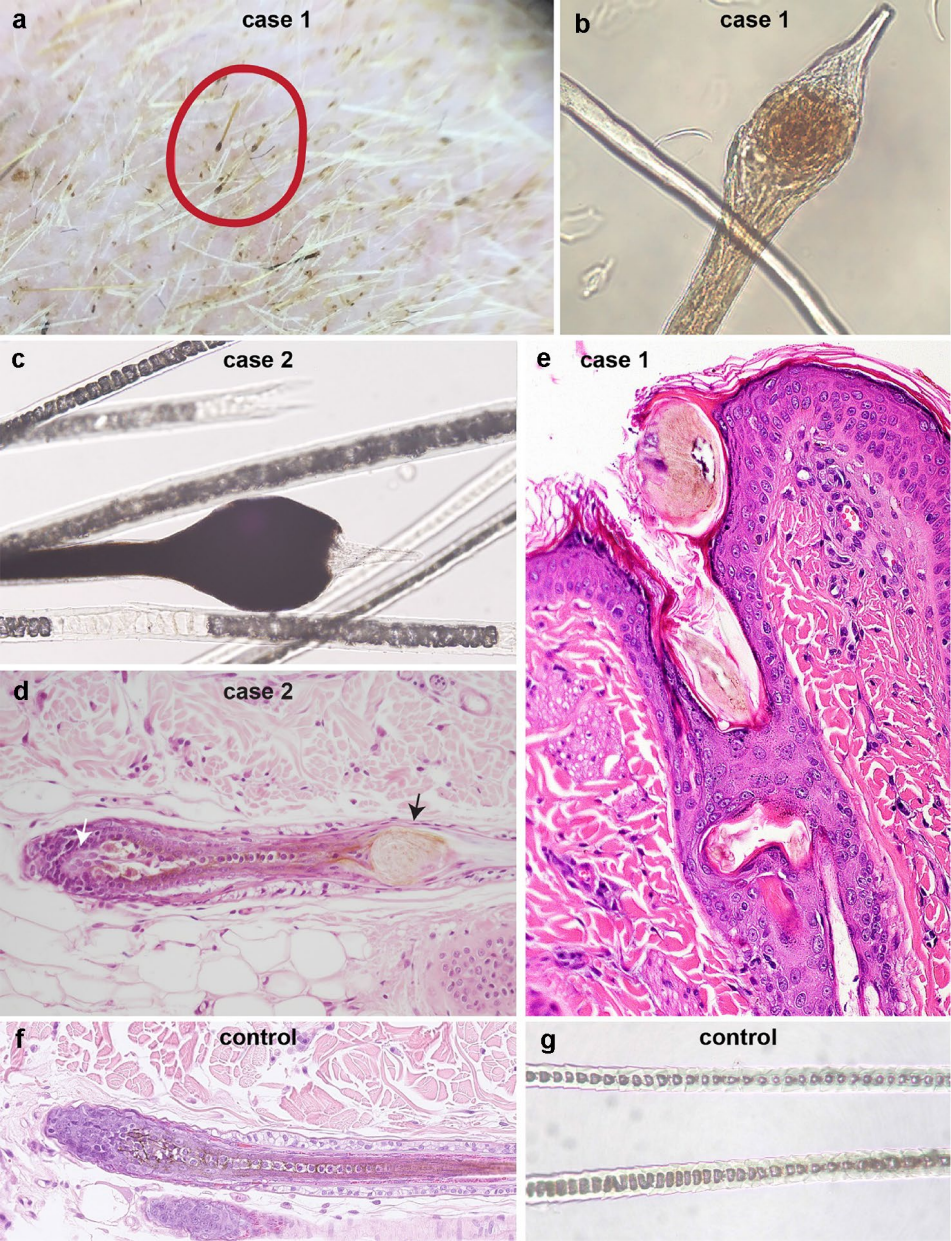
Clinical and histological features of the lanceolate hair shafts and follicles of cases no. 1 and no. 2 (**a**–**e**). **a** The Dermascope image shows hair with irregular thickenings of the hair shafts (red circle). **b**–**c** Trichogram of hair with lance-head shaped distal ends. **d** The histological image of an anagen hair follicle shows a bulbous or lance-head shaped swelling of the already fully cornified hair shaft (black arrow) just distal to the melanogenic zone at the border of inferior portion and isthmus. This is associated with individual cells or small cell clusters in the precortex and premedulla (white arrow) that are enlarged, rounded, and have abundant pale cytoplasm, suggesting that they are undergoing degenerative changes. **e** The bulbous swelling of the hair shaft is located close to the orifice. The cortex of the hair shaft is fragmented and broken hair shaft fragments are oriented horizontally. **f** Histology of a feline control hair follicle. **g** Trichogram of a feline control hair

Case no. 1 was a female random-bred DSH cat, born in 2019 on a farm in Switzerland. An unusual generalized moderate-to-severe hypotrichosis was observed at the first check-up examination at 3 months of age. The cat was otherwise in good general health. The owner reported that a maternal half-sibling of this cat had been born naked and died within a few days after birth. The hair loss in case no. 1 symmetrically affected the convex pinnae, parts of the face, the back, and the legs including the dorsal paws (Fig. 1a, b). The cat was mildly pruritic. Dermatophytosis and infestation with ectoparasites as underlying cause for the alopecia were excluded clinically and by treatment with Fluralaner (Bravecto(R), MSD Animal Health GmbH, Switzerland) and topical enilconazole (Imaverol(R), Covetrus AG, Switzerland). The trichogram revealed broken and split hair shafts. The hair shafts presented with severe bulbous swellings. These swellings were present anywhere in the hair shaft but most often close to the tip (Fig. 2a, b).

In 2010, a 1.5 year old male castrated DSH cat (case no. 2) from a local shelter in Germany was presented with progressive alopecia of the dorsum, the plantar, and palmar surfaces of the limbs, the convex pinnae, and most of the face, as previously published (Fig. 1c, d) (Rostaher et al. 2021). In addition to body hairs being affected, the vibrissae were short and broken. Macroscopic evaluation of the skin surface and microscopic examination of trichograms revealed many short, broken hair shafts, some of which had bulbous or lance-head shaped ends (Fig. 2c). All of the cat’s littermates showed similar skin lesions.

The histopathological evaluation revealed that the number and size of the hair follicles was normal and the presence of hair shafts in all hair follicles. Most hair follicles were in anagen. Numerous, but not all hair shafts were dystrophic. The dystrophy was characterized by severe well circumscribed thickening of the hair shafts starting above the melanogenic zone (Fig. 2d). In addition, there was loss of a ladder-like pattern of pigment distribution. In Fig. 2e, the swelling of the hair shaft was present more distally in the infundibulum. This reflects most likely the outgrowing of the bulbous swelling and represents the lance-head shaped hair tip. Additionally, the dystrophy was characterized by an irregular outer contour of the hair shafts, fragmentation within the cortex and the cuticle, and dense eosinophilic cornified material that surrounded the hair shafts. In some infundibula, the fragmented hair shafts were oriented horizontally. Furunculosis was present in one biopsy. Sebaceous and sweat glands were normal. The epidermis was moderately hyperplastic and covered by a small amount of laminar orthokeratotic keratin (Fig. 2e).

### Genetic analysis

Given the results of the clinical and histopathological analysis, *DSG4* was considered the primary functional candidate gene for the observed phenotype in both cases. To characterize the underlying causative genetic variant, we sequenced the genome of the affected cats and searched for private variants in *DSG4*. Since the cats were not known to be related, we hypothesized that their phenotypes were due to independent pathogenic variants. We therefore searched for private variants in each of the affected cats’ genomes individually by comparing them to the genomes of 61 other cats (Table 2, Table S1, Table S2).

**Table 2:** Results of variant filtering in the affected cats against 61 control genomes

Filtering step	Variants case no. 1	Variants case no. 2
All variants	5,955,464	6,162,272
Private variants	26,179	26,624
Protein-changing private variants	76	93
Protein-changing private variants in *DSG4*	1	1

Only homozygous variants are reported

This analysis identified a single homozygous private protein-changing candidate variant in *DSG4* in each of the cases. The two variants can be designated as ChrD3:55,315,010del (FelCat9.0) for case no. 1 and ChrD3:55,336,127del (FelCat9.0) for case no. 2. Both variants represented 1 bp deletions, causing frameshifts and resulting in premature stop codons (Table 3). The variants truncate 98% and 43% of the open-reading frame, respectively.

**Table 3:** Variant designations of the identified *DSG4* frameshift deletions

Cats	HGVS variant designations		
	Genomic (FelCat9.0)	mRNA (XM_019815116.1)	Protein (XP_019670675.1)
Case no. 1	ChrD3:55,315,010del	c.76del	p.Ile26Leufs*4
Case no. 2	ChrD3:55,336,127del	c.1777del	p.His593Thrfs*23

**Fig. 3:**
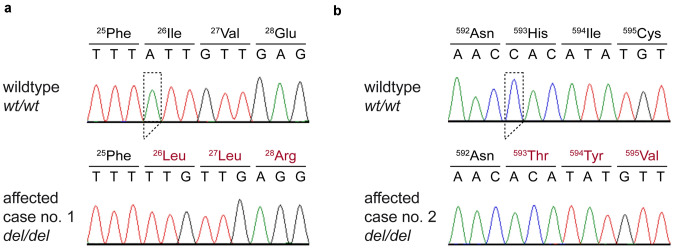
Details of the **a** *DSG4*:c.76del and **b** *DSG4*:c.1777del variants. Representative electropherograms of a control and the affected cats are shown. The amino acid translations of the wild-type and mutant alleles are indicated

We confirmed the presence of the *DSG4* frameshift variants by Sanger sequencing (Fig. 3) and genotyped a cohort of 46 unaffected cats from different breeds for both variants. The affected cats carried one frameshift allele in a homozygous state and were homozygous wild-type at the variant of the other case. All other non-affected cats were homozygous for the wild-type allele at both variants (Table 4).

**Table 4:** Genotype–phenotype association of the *DSG4* variants with hair shaft dystrophy in cats

Cats	c.76del			c.1777del		
	wt/wt	wt/del	del/del	wt/wt	wt/del	del/del
Case no. 1	–	–	1	1	–	–
Case no. 2	1	–	–	–	–	1
Controls (*n* = 46)	46	–	–	46	–	–

## Discussion

In this study, we investigated two unrelated DSH cats affected with hair shaft dystrophy. One of the two investigated cats (case no. 2) was part of a previous study about the clinical, histological, and ultrastructural features of the disease, which revealed significant similarity to *Dsg4*^*lah*^ and *Dsg4*^*lahJ*^ mutant mice (Rostaher et al. 2021). The other case (case no. 1) was an unrelated DSH cat that presented with a similar clinical and histopathological phenotype.

We sequenced the genomes of both cats and filtered for private homozygous variants in our primary functional candidate gene *DSG4*. This analysis identified independent private variants in each of the cases. Both variants represented frameshift deletions predicted to result in true null alleles. The finding of independent loss-of-function variants in the same gene in cats with comparable phenotype represents a very strong evidence for the causality of these variants.

*DSG4* has been identified as the fourth and last member of the desmoglein family (Whittock and Bower 2003). In human anagen hair follicles, *DSG4* is expressed in the precortex, cortex, and lower cuticle of the hair shaft. It is additionally expressed in the inner root sheath cuticle above the bulb region (Bazzi et al. 2006). As component of the desmosomes, *DSG4* is involved in linking the intracellular network of keratins of a trichocyte to the intermediate filaments of the neighbouring trichocytes. This intra- and inter-cellular network of keratins and desmosomes provides the hair shaft with its tensile strength and elasticity (Bazzi et al. 2009). It also plays a central role in coordinating cellular dynamics in the lower hair follicle during the switch from proliferation to differentiation (Kljuic et al. 2003). The mutant cats had a prominent hair shaft defect characterized by a bulbous swelling distal to the melanogenic zone. This probably leads to an increased fragility and the formation of a lance-head shaped hair tip (Rostaher et al. 2021).

Variants in *DSG4* have previously been described in mice and rats with lanceolate hair phenotype, and in human patients with localized autosomal recessive hypotrichosis, as well as monilethrix-like hypotrichosis (Kljuic et al. 2003; Jahoda et al. 2004; Moss et al. 2004; Nagasaka et al. 2004; Rafiq et al. 2004; Messenger et al. 2005; John et al. 2006; Schaffer et al. 2006; Shimomura et al. 2006; Zlotogorski et al. 2006; Wajid et al. 2007). We assume that loss of *DSG4* function leads to an incorrect formation of the desmosomes resulting in impaired adhesion between neighbouring trichocytes and a defective hair shaft formation in the affected cats.

In summary, we identified two likely disease-causing variants in *DSG4* in cats with a recessive hair shaft dystrophy. The hair phenotype of the affected cats was comparable to the phenotype in lanceolate hair mice. Our findings therefore corroborate the relevance of desmoglein 4 for correct hair shaft formation and the known genotype–phenotype correlations from other species. The affected cats were homozygous for the mutant alleles, indicating that inbreeding contributed to the observed genodermatoses. To the best of our knowledge, this study represents the first report of pathogenic *DSG4* variants in domestic animals.

## Supplementary Information

Below is the link to the electronic supplementary material.

Supplementary file1 (XLSX 13 KB)

Supplementary file2 (XLSX 9596 KB)

## Data Availability

The genome sequence data were submitted to the European Nucleotide Archive (ENA). All accession numbers are listed in Table S1.
